# Functional bacterial amyloid increases *Pseudomonas* biofilm hydrophobicity and stiffness

**DOI:** 10.3389/fmicb.2015.01099

**Published:** 2015-10-07

**Authors:** Guanghong Zeng, Brian S. Vad, Morten S. Dueholm, Gunna Christiansen, Martin Nilsson, Tim Tolker-Nielsen, Per H. Nielsen, Rikke L. Meyer, Daniel E. Otzen

**Affiliations:** ^1^Interdisciplinary Nanoscience Centre, Aarhus UniversityAarhus, Denmark; ^2^Center for Microbial Communities, Aalborg UniversityAalborg, Denmark; ^3^Department of Biomedicine-Medical Microbiology and Immunology, Aarhus UniversityAarhus, Denmark; ^4^Department of Immunology and Microbiology, Costerton Biofilm Center, Faculty of Health and Medical Sciences, University of CopenhagenCopenhagen, Denmark

**Keywords:** amyloid, *Pseudomonas*, biofilm, AFM, force spectroscopy, Young's modulus, contact angle

## Abstract

The success of *Pseudomonas* species as opportunistic pathogens derives in great part from their ability to form stable biofilms that offer protection against chemical and mechanical attack. The extracellular matrix of biofilms contains numerous biomolecules, and it has recently been discovered that in *Pseudomonas one* of the components includes β-sheet rich amyloid fibrils (functional amyloid) produced by the *fap* operon. However, the role of the functional amyloid within the biofilm has not yet been investigated in detail. Here we investigate how the *fap*-based amyloid produced by *Pseudomonas* affects biofilm hydrophobicity and mechanical properties. Using atomic force microscopy imaging and force spectroscopy, we show that the amyloid renders individual cells more resistant to drying and alters their interactions with hydrophobic probes. Importantly, amyloid makes *Pseudomonas* more hydrophobic and increases biofilm stiffness 20-fold. Deletion of any one of the individual members of in the *fap* operon (except the putative chaperone FapA) abolishes this ability to increase biofilm stiffness and correlates with the loss of amyloid. We conclude that amyloid makes major contributions to biofilm mechanical robustness.

## Introduction

Most bacteria are able to form biofilms or “microbial cities,” which renders the bacteria resistant to conventional antibiotics as well as mechanical and chemical attack. *Pseudomonas* strains in particular show a remarkable ability to form biofilm in a wide range of environments, allowing, e.g., *P. aeruginosa* to colonize lungs as an opportunistic pathogen in cystic fibrosis (Alhede et al., 2014). Biofilm protects bacterial communities by encasing them within a matrix of extracellular polymeric substances (EPS) (Rasamiravaka et al., 2015). This matrix stabilizes the establishment of microbial cells on a surface, and is likely to be a major factor facilitating most microbial infections (Costerton et al., 1999). EPS consists of different biomolecules, including polysaccharides, extracellular DNA (eDNA) and proteins, leading to a highly hydrated and polar structural scaffold (Flemming and Wingender, 2010). There has been much focus on the role of polysaccharides and eDNA, which are both involved in early stages of biofilm formation (Whitchurch et al., 2002; Vasseur et al., 2005). Polysaccharides contribute to structural stability and protection in addition to helping bind and retain water and nutrients (Sutherland, 2001), while eDNA maintains coherent cell alignments (Gloag et al., 2013) and also serves as a source of nutrients during starvation (Mulcahy et al., 2010). In contrast, the role(s) of the protein components remain less well studied. Nevertheless, there is increasing evidence that proteins also play a major role in the build-up of biofilm.

A few years ago, we discovered an operon in a *Pseudomonas* sp. which we dubbed *fap* (Functional Amyloid in *Pseudomonas*) (Dueholm et al., 2010) and which upon overexpression in *E. coli* led to the formation of significant amounts of biofilm. This operon leads to the formation of amyloid fibrils, i.e., long and thin structures extending from the surface of the bacterial outer membrane which are arranged in the classical amyloid pattern with β-strands stacked orthogonal to the main fibril axis. The *fap* operon contains 6 genes (*fapA-F*) that encode the respective Fap proteins. Of these, FapC is the main fibril monomer, while FapB and FapE only make up a small percentage of purified fibrils (Dueholm et al., 2013a). FapB can take over as the main amyloid component *in vivo* if FapA is knocked out (Dueholm et al., 2013a), implying that FapA has a more regulatory role. Sequence analysis suggests that FapF is a β-barrel membrane protein and FapD a peptidase (Dueholm et al., 2013a). While such amyloid structures are typically associated with neurodegenerative diseases such as Alzheimer's and Parkinson's (Otzen, 2012), it is increasingly clear that they also have a vital and beneficial role to play in bacteria in general (Chapman et al., 2002; Dueholm et al., 2012). Amyloid is found widespread in the bacterial kingdom (Larsen et al., 2007, 2008) and we have very recently reported their existence in *Archaea* (Dueholm et al., 2015). Though not as widespread as the operon which in *E. coli* produces amyloid called curli, the *fap* operon (whose amyloid product we refer to as Fap amyloid) is found not just in *Pseudomonas* species (belonging to the Gammaproteobacteria) but also among Beta- and Deltaproteobacteria (Dueholm et al., 2013b). Bacterial amyloids serve a variety of functions, and so far the structural role appears to dominate, either in the form of self-associating fibrils or possibly even as direct components of the cell wall (Jordal et al., 2009; Dueholm et al., 2015). In addition to being able to associate laterally and entangle with each other, fibrils are also able to bind small metabolites such as quorum-sensing molecules which are instrumental in bacterial intercellular communication and biofilm build-up (Seviour et al., 2015). This makes them potential reservoirs to retain metabolites and nutrients within the biofilm, which is a very useful feature under conditions of e.g., turbulent flow in aquatic environments like rivers or wastewater treatment plants.

Hitherto it has not been addressed to what extent amyloid affects the mechanical properties of the biofilm. We would expect amyloid to play a significant role, given that amyloid can show tensile strength comparable to steel on a weight-to-weight basis, making them high-performance biomaterials (Knowles et al., 2007). Biofilms themselves need to be sufficiently robust to withstand mechanical insults (typically shearing forces Tiirola et al., 2009) while being soft enough to accommodate bacterial movement and proliferation (Tolker-Nielsen et al., 2000). Biofilm is generally robust toward chemical treatment; in fact, Fe^3+^ can increase biofilm elasticity by a factor of ~500 (from ca. 1 kPa to 0.5 MPa), probably through interactions with negatively charged groups in the EPS, though this is countered by the addition of the Fe-chelator citric acid (Lieleg et al., 2011). Here we use atomic force microscopy (AFM) to probe the effect of functional amyloid on the robustness and stiffness of the amyloid-producing *Pseudomonas* sp. UK4 (UK4) found within the *P. fluorescens* group (Dueholm et al., 2010). We show that amyloid makes the cells much more resistant toward drying out, increases the hydrophobicity of the biofilm surface and increases biofilm stiffness 20-fold. Deletion of individual genes in the *fap* operon highlights different contributions of individual components. We conclude that bacterial amyloid makes a major contribution to the build-up of biofilm's mechanical robustness.

## Materials and methods

### Materials

Unless otherwise stated, all chemicals were from Sigma-Aldrich. The UK4 *fap* operon overexpression vector pMMB190Tc-UK4fap (pFap) was obtained from an earlier study (Dueholm et al., 2013a).

### Cloning of the UK4Δ*fap* mutant

To remove the entire *fap* operon (*fapA-fapF)* from UK4, a knockout fragment containing a gentamicin (Gm) resistance cassette was generated by PCR overlap extension essentially as described by Choi and Schweizer (2005) and Dueholm et al. (2013a). Briefly, primers (see Table 1) were used to amplify chromosomal regions upstream (UK*fap*AUpF-GWL/UK*fap*AUpR-GM) and downstream (UK*fap*FDnF-GM/UK*fa*pFDnR-GWL) of *fapA*-*fapF*, and to amplify a gentamicin resistance cassette (Gm-F/Gm-R) from plasmid pPS856 (Hoang et al., 1998). The PCR fragments were fused together and amplified with primers GW-attB1 and GW-attB2, incorporating the *att*B1 and *att*B2 recombination sites at either end of the knockout cassette. Using the Gateway cloning system (Invitrogen), the resulting knockout fragment was first transferred by the BP reaction into pDONR221, generating entry plasmid pDONR221-ΔUK4*fap*, and subsequently transferred by the LR reaction into pEX18ApGW generating the knockout plasmid pEX18ApGW-ΔUK4*fap*. This plasmid was then transferred into UK4 by two-parental mating using the donor strain *E. coli* S17-1 with selection on *Pseudomonas* isolation agar plates supplemented with 30 μg/mL gentamicin. Resolution of single crossover events was achieved by streaking on 5% sucrose plates via the counter-selectable *sacB* marker on the knockout plasmid. The mutant construction was confirmed by PCR analysis.

**Table 1:** **Bacteria, plasmids and primers used in this study**.

**Species and strain**	**Characteristics/Sequence**	**References**
***Escherichia coli***		
Mach1	Used for rutine subcloning	Invitrogen
S17-1	*pro thi recA hsdR (r− m+)* Tp^*r*^ Sm^*r*^ Km^*r*^ Δ RP4-2-Tc::Mu-Km::Tn7	Simon et al., 1983
***Pseudomonas*** **sp. UK4**		
WT	Wildtype	Dueholm et al., 2010
Δ*fap*	*fapA-fapF* inactivated in PAO1 by allelic displacement with a gentamicin-resistance cassette using pEX18ApGW-UK4*fap*; Gm^*r*^	This study
pVC	Δ*fap* with pMMB190Tc	This study
pFap	Δ*fap* with pMMB190Tc-UK4*fap*	This study
pFapΔA	Δ*fap* with pMMB190Tc-UK4*fap*Δ*fap*A	This study
pFapΔB	Δ*fap* with pMMB190Tc-UK4*fap*Δ*fap*B	This study
pFapΔC	Δ*fap* with pMMB190Tc-UK4*fap*Δ*fap*C	This study
pFapΔD	Δ*fap* with pMMB190Tc-UK4*fap*Δ*fap*D	This study
pFapΔE	Δ*fap* with pMMB190Tc-UK4*fap*Δ*fap*E	This study
pFapΔF	Δ*fap* with pMMB190Tc-UK4*fap*Δ*fap*F	This study
**PLASMIDS**		
pMMB190Tc	pMMB190 Δ*bla tet*(Tc^*R*^) P*_*lacUV*5_* controlled expression	Dueholm et al., 2013a
pMMB190Tc-UK4*fap*	pMMB190Tc containing the complete UK4 *fap* operon	Dueholm et al., 2013a
pMMB190Tc-UK4*fap*ΔA	pMMB190Tc-UK4*fap* with a deletion of *fapA*	This study
pMMB190Tc-UK4*fap*ΔB	pMMB190Tc-UK4*fap* with a deletion of *fapB*	This study
pMMB190Tc-UK4*fap*ΔC	pMMB190Tc-UK4*fap* with a deletion of *fapC*	This study
pMMB190Tc-UK4*fap*ΔD	pMMB190Tc-UK4*fap* with a deletion of *fapD*	This study
pMMB190Tc-UK4*fap*ΔE	pMMB190Tc-UK4*fap* with a deletion of *fapE*	This study
pMMB190Tc-UK4*fap*ΔF	pMMB190Tc-UK4*fap* with a deletion of *fapF*	This study
pDONR221	Gateway donor vector; Km^*r*^	Invitrogen
pEX18ApGW	Gateway compatible gene replacement vector; Suc^*s*^, Amp^*r*^	Choi and Schweizer, 2005
pPS856	0.83 kb blunt-ended SacI fragment from pUCGM ligated into the EcoRV site of pPS854; Amp^*r*^, Gm^*r*^	Hoang et al., 1998
pDONR221-ΔUK4*fap*	*fapA-fapF* entry clone; Km^*r*^, Gm^*r*^	This study
pEX18ApGW-ΔUK4*fap*	UK4*fap* knockout vector; Suc^*s*^, Amp^*r*^, Gm^*r*^	This study
**PRIMERS**		
UK*fap*AUpF-GWL	5′-TACAAAAAAGCAGGCTAAGATCA GCCATGACCGCG	This study
UK*fap*AUpR-GM	5′- TCAGAGCGCTTTTGAAGCTAA TTCGTGAGAGTCGCCACGGCAG	This study
UK*fap*FDnF-GM	5′-AGGAACTTCAAGATCCCCAATT CGCATTACCTACGCGCGCTATGA	This study
UK*fap*FDnR-GWL	5′- TACAAGAAAGCTGGGTAGGCT GAAGGTGAAGTCGGG	This study
Gm-F	5′- CGAATTAGCTTCAAAAGCGCT CTGA	Choi and Schweizer, 2005
Gm-R	5′- CGAATTGGGGATCTTGAAGTT CCT	Choi and Schweizer, 2005
GW-*attB*1	5′- GGGGACAAGTTTGTACAAAAA AGCAGGCT	Choi and Schweizer, 2005
GW-*attB*2	5′- GGGGACCACTTTGTACAAGAA AGCTGGGT	Choi and Schweizer, 2005
pMMB190-UpF	5′-ACACAGGAAACTAGGCAC	This study
pMMB190-DnR	5′- AAATCTTCTCTCATCCGCC	This study
LmarkR-UK4fapA-UpR	5′-AGGAACTTCAAGATCCCCAATT CGAGCCTTGGTTGAGAGTCG	This study
LmarkR-UK4fapB-UpR	5′-AGGAACTTCAAGATCCCCAATT CGAGCAGCCAAGAACGGTGA	This study
LmarkR-UK4fapC-UpR	5′-AGGAACTTCAAGATCCCCAATT CGGGTTTGAGAGCCATTGTAG	This study
LmarkR-UK4fapD-UpR	5′-AGGAACTTCAAGATCCCCAATT CGCTGGAAATAAAAAGGGCCT	This study
LmarkR-UK4fapE-UpR	5′-AGGAACTTCAAGATCCCCAATT CGAACGGGAAGTGTTCATCAT	This study
LmarkR-UK4fapF-UpR	5′-AGGAACTTCAAGATCCCCAATT CGTTAAACAGACAACGGCACG	This study
LmarkF-UK4fapA-DnF	5′-CGAATTGGGGATCTTGAAGTT CCTTGGCACCGTTGAATAACA	This study
LmarkF-UK4fapB-DnF	5′-CGAATTGGGGATCTTGAAGTT CCTGAACCGCATGGCTAACAC	This study
LmarkF-UK4fapC-DnF	5′-CGAATTGGGGATCTTGAAGTT CCTGCAAAGCAACACCCTCAC	This study
LmarkF-UK4fapD-DnF	5′-CGAATTGGGGATCTTGAAGTT CCTCGCCAAGACCAAAATGAAC	This study
LmarkF-UK4fapE-DnF	5′-CGAATTGGGGATCTTGAAGTT CCTACTGCAATCTGGATCAACT	This study
LmarkF-UK4fapF-DnF	5′-CGAATTGGGGATCTTGAAGTT CCTACCTGACAATTGTGCCCA	This study
EcoRI-UK4*fap*F	5′-CACTGAATTCGCTTCTGCT CTATTCCTCAC	Dueholm et al., 2013a
HindIII-UK4*fap*R	5′-CACTAAGCTTGCGCAGCGGT TTTAGAAGT	Dueholm et al., 2013a

### Construction of pFap single gene knockout derivatives

Single gene knockout derivatives of pFap were obtained by PCR overlap extension (Horton et al., 2013). Briefly, primers were used to amplify the regions spanning the upstream region of the *fap* operon to the 5′-terminal of the target gene (pMMB190-UpF/LmarkR-UK4*fap*X-UpR) and the 3′-terminal of the target gene to the downstream region of the *fap* operon (LmarkF-UK4*fap*X-DnF and pMMB190-DnR) in pMMB190Tc-UK4*fap*. The PCR fragments were fused together using an incorporated reverse complemented sequence and amplified with the EcoRI-UK4fapF and HindIII-UK4fapR primers (Dueholm et al., 2013a). The final PCR fragments, which contained the *fap* operon with the individual genes disrupted, were subcloned into pCR4-TOPO vectors (Life technologies). Inserts for cloning were obtained by digestion of the subcloned vectors with FastDigest EcoRI/HindIII (Thermo Scientific) followed by gel purification using the UltraClean 15 DNA kit (MO-BIO, Carlsbad, CA). The expression vector pMMB190Tc was prepared for ligation using the same restriction enzyme combinations and purified as above. Ligations were done using T4 DNA ligase (Life technologies) according to the manufacturers' recommendation and the resulting vectors were validated by shotgun sequencing of the whole plasmids (Macrogen).

### Transformation of UK4Δ*fap*

Electrocompetent UK4Δ*fap* cells were prepared as previously described (Choi et al., 2006). Fifty microliter of electrocompetent cells were mixed with 2 μL plasmid (400 ng/μL) and 40 μL of the suspension was transferred to a room temperature 1 mm gap electroporation cuvette. A pulse of 1.40 kV was applied using a MicroPulser electroporator (BioRad). One milliliter room temperature LB medium was immediately applied and the samples transferred to a 15 mL tube. The transformation was incubated (28°C, 200 rpm, 2 h) and 100 μL were plated out on a LB agar plate containing 50 μg/mL tetracycline. Transformed colonies were picked after 3 days.

### Transmission electron microscopy

UK4Δ*fap* transformed pFap and single gene knockout derivatives were grown overnight (26°C, 200 rpm) in colony factor antigen (CFA) medium (10 g/L casein hydrolysate (Fluka), 1.5 g/L yeast extract (Sigma), 50 mg/L MgSO_4_, and 5 mg/mL MnCl_2_, adjusted to pH 7.4 with NaOH) containing 50 μg/mL tetracycline. These starter cultures were used to inoculate 10 mL of CFA with 50 μg/mL tetracycline and 1 mM isopropyl β–D-1-thiogalactopyranoside (IPTG) to OD_600_ 0.05. The samples were grown at 26°C with shaking at 200 rpm. Samples for TEM were collected at the stationary phase. Grids were washed in two drops of MilliQ water and stained with 1% phosphotungstic acid (pH 6.8) and blotted dry on filter paper. Samples were viewed with a JEOL 1010 transmission electron microscope.

### Congo red binding assay and thioflavin T (ThT) staining

The same strains used for TEM analysis were grown on Congo Red indicator plates composed of CFA medium supplemented with 20 μg/mL Congo red and 10 μL/mL Coomassie brilliant blue G-250 solidified with 2% agar (AppliChem). One micrometer IPTG was used to induce production of Fap amyloid in overexpressing mutants. Growth was at 26°C. ThT was dissolved in 10 mM TRIS buffer at pH 8.0 at concentration of 7.5 μM. Bacterial colonies from agar plates were stained with ThT for 15 min before observation under epi-fluorescence microscope (Axiovert 200 M, Zeiss, Germany) with Zeiss filterset 06 (excitation 431–441, emission 470 LP).

### Growth of UK4 for AFM studies

UK4 and derivative were cultured in CFA medium (26°C, 180 rpm) or on CFA agar. Forty microgram per milliliter tetracycline was used to select for mutants carrying the pMMB190Tc plasmids. For this assay, leaky expression of cloned *fap* operons was sufficient and no IPTG was used (Dueholm et al., 2013a).

### AFM imaging

Bacteria were grown in CFA medium (40 h, 26°C, 180 rpm), harvested and washed three times by centrifugation (5000 rpm, 3 min) followed by resuspension in MilliQ water. This was sufficient to detach individual cells for AFM imaging. A 5 uL droplet of bacterial suspension was placed on freshly cleaved mica and dried in air for 15–30 min and immediately imaged by AFM in air. In this way, morphological changes due to drying over time were minimized. For imaging of WT cell aggregates, extra precautions were taken: A cell aggregate was identified and tracked under optical microscope during the drying process and then imaged afterwards. This is necessary because single cells tend to be forced to come close and form fake aggregates after drying out.

A NanoWizard II (JPK Instruments, Germany) combined with an inverted optical microscope (Axiovert 200 M, Zeiss, Germany) was used for all AFM measurements. Tapping mode imaging was conducted with OMCL-AC160TS (Olympus) cantilevers. For each bacterial strain, at least three images at random locations were obtained for each sample, and measurements were repeated with independently grown cultures to confirm that similar morphologies were observed.

### AFM force spectroscopy on single cells

AFM force spectroscopy on single cells was performed in the following way: a colloid glued on an AFM cantilever (see below) was approached on single cells immobilized on glass slides, kept at force set point for a specific amount of time, and then retracted. The deflection of the cantilever and displacement of the cantilever as a result of the piezo movement was recorded. The deflection-displacement curves were converted to force-distance curves (force curves hereafter) after calibration of cantilever sensitivity and spring constant. Sensitivity was determined by recording force curves on glass slides and spring constant was calibrated using the thermal tuning method (Hutter and Bechhoefer, 1993).

Tipless cantilevers with a nominal spring constant of 0.03 N/m (HQ:CSC38/TIPLESS/NO AL, MikroMasch Europe) were used for force spectroscopy. To attach a colloid, a tipless cantilever was approached on a small drop of UV curable adhesive (LOCTITE, part no. 17944) on glass slide and retracted. One to two additional approaches on clean glass slide were conducted to remove excessive glue, and the cantilever was then approached on a 5.6 μm silica colloid (Microparticles GmbH) under optical microscope and retracted after 1 min. One minutes UV irradiation with the built-in mercury lamp was used to cure the glue, leading to formation of a colloid prober. The colloid probe was cleaned by UV/Ozone for 20 min to yield a hydrophilic surface. To make the colloid hydrophobic, the cleaned colloid probe was incubated in anhydrous toluene with 1% v/v (3,3,3-Trifluoropropyl)trimethoxysilane overnight, and washed with toluene and trichloromethane.

For successful force spectroscopy on single cells, cells must be firmly attached on the substrate. To achieve this, glass slides were coated with wet adhesive polydopamine. Four milligram per milliliter dopamine in 10 mM Tris buffer pH 8.5 were incubated on glass slides for 1 h, after which the glass slides were washed with MilliQ water.

Bacterial samples were prepared almost the same way as for AFM imaging, except that cells were washed with PBS instead of water to avoid stressing the cells. This was sufficient to obtain individual cells of the WT strain. As pFap cells are mostly severely flocculated, 30 s sonication (Branson B5510, 135W) was used to break aggregates and obtain a reasonable amount of single cells for measurements. A 5 μm cell suspension in PBS was placed on a coated glass slide for 5 min and washed extensively with PBS to remove unattached cells. A 100 μL drop of PBS was added for liquid AFM force spectroscopy. A colloid probe was calibrated on a glass slide, approached at 4 μm/s on an immobilized single cell for 5 s at 1 nN setpoint, and then retracted. The positioning of the colloid probe was done with optical microscope under 40X long working distance objective lens. The process can be facilitated by using DirectOverlay on NanoWizard II, which allows identification of the tip of the colloid and automation of the measurement on multiple cells. Successful approaches on single cells were seen as a gradual increase of repulsive force after contact point, whereas approaches on coated glass gave a sharp linear increase after contact. More than 10 force curves were recorded on each cell, and at least 10 cells were measured.

### AFM force spectroscopy on cell aggregates

The same procedure for making samples for AFM imaging was used. Cell aggregates were first imaged by AFM imaging in air to aid the selection of positions of measurement, and then added with PBS to perform force spectroscopy with colloid probe. The drying and rehydration process ensured the firm attachment of cell aggregates in liquid and therefore no coating was needed. More than 10 force curves were recorded at each position, and at least 5 positions were measured.

### AFM nanoindentation on biofilm

Biofilm of WT and the 8 different mutants was grown on 20 mm square glass coverslips placed in 12-well plates with CFA medium inoculated with colonies from agar plates. The plates were incubated at 26°C for 40 h with no shaking. A glass microbead (nominal size 59.2 μm, G4649-10G, Sigma-Aldrich) was glued to a tipless AFM cantilever using the same procedure as for the colloid probe. The actual size of the glass microbead was determined from optical images. To avoid contamination of the microbead probe during measurement, an antifouling coating was applied to the UV/ozone cleaned microbead probe by incubating in 100 μg/mL PLL (20 kDa)-g(4.0)-PEG (5 kDa) (Susos AG, Dubendorf, Switzerland) in 10 mM HEPES for 2 h and subsequent washing with MilliQ water. Ten force curves were recorded on each of the 5 locations, and measurements were repeated once on different samples. Measurement locations were selected in the area where the biofilm is estimated to be more than 20 μm thick by reading the movement of the optical stage from the bottom to the top.

To calculate the Young's modulus (*E*) of biofilm from the indentation force curves, Sneddon's modification of Hertz model of contact mechanics was employed (Hertz, 1882; Sneddon, 1965). When a stiff sphere indents a soft planar sample, the loading force *F* is related to indentation depth δ by:

(1) $$\begin{array}{l} F=\;\frac{4}{3}\frac{E}{\left(1-\nu^{2}\right)}R\delta^{3∕2}, \end{array}$$

where *R* is the radius of the sphere and ν is the Poisson ratio (assumed to be 0.5) of the sample. The Hertz model requires several assumptions to be met: the adhesion force between sphere and sample is negligible; the sample is homogeneous and semifinite (i.e., the indentation is small compared to the thickness of the film) and the sample undergoes small strains within the elastic limit. While these assumptions are almost impossible to be met at the same time for most biological samples, the model was proven to be useful for soft biological samples when indentation was limited to a small range (<10%) compared to the sample thickness and the loading speed is low enough to minimize contribution from plastic deformation and hydrodynamic forces. The indentation depth in the current study was limited to 2 μm and loading speed was set at 1 μm. The indentation curves were fit by Hertz model using JPK Data Processing software, giving the contact points and Young's moduli (*E*) of the samples.

The plastic properties of the samples were characterized by the plasticity index ψ_*P*_ in the form:

(2) $$\begin{array}{l} \psi_{P}=\;\frac{A_{1}}{A_{1}+A_{2}}, \end{array}$$

where *A*_1_ and *A*_2_ are the energy of plastic deformation and elastic recovery, respectively, calculated from the area in force curves.

### Hydrophobicity of bacteria

Bacterial cells were harvested and washed in water as described above. A bacterial lawn was coated on a glass slide by drying a 100 μL droplet of bacterial suspension overnight. Hydrophobicity of bacteria was characterized by dynamic contact angle measurement using a Drop Shape Analyzer DSA100 (Krüss GmbH, Germany). A water droplet was formed on the bacterial lawn and manually expanded by syringe injection. The change of the contact angle over time was monitored by video recording and submitted to frame by frame analyzation, and contact angles were plotted against time. Due to the high surface heterogeneity, contact angles rose, and fell in repetitive patterns over time. The highest peak of contact angles, which reflected the state the liquid was about to wet new surface, was determined as advancing contact angles. Three samples were tested for each strain.

### Statistics

Data were presented as mean ± SD. Student's *t*-test was used for comparing two groups of data, and significant difference was claimed when *P* < 0.05.

## Results

### Staining confirms the presence of amyloid in the pseudomonas strain overexpressing pFap

We started out by creating appropriate derivatives of *Pseudomonas* sp. UK4 to allow for proper comparison. We constructed a whole *fap* operon deletion mutant (Δ*fap)*. This involves deletion of the open reading frames for the major amyloid component FapC as well as the ancillary proteins FapA-B and FapD-F (Dueholm et al., 2010, 2013b). Since UK4 wildtype (WT) only produces amyloid to a small degree, we also cloned the *fap* operon overexpression vector pMMB190Tc-UK4*fap*, where expression was under the control of the lacUV5 promoter, into the Δ*fap* strain. This led to the pFap strain.

**Figure 1:**
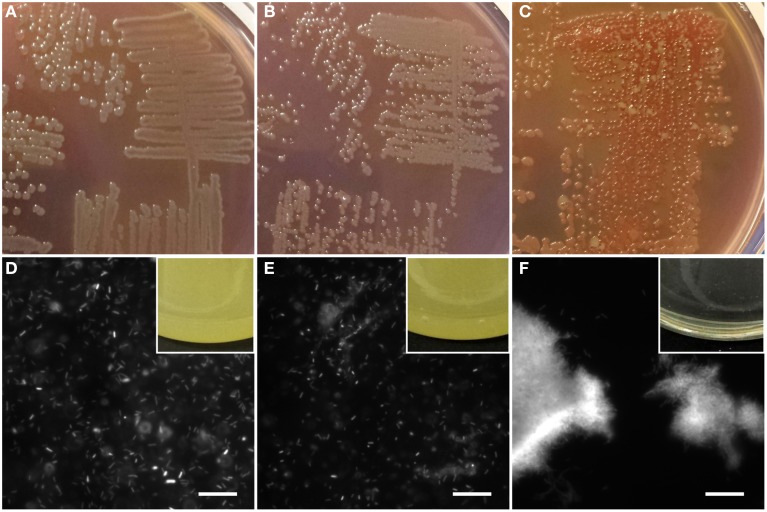
**Identification of amyloid production by staining**. Bacterial colonies of WT **(A)**, Δ*fap* **(B)**, and pFap **(C)** grown on Congo red-CFA agar plates. Fluorescent images of liquid cultures of WT **(D)**, Δ*fap* **(E)**, and pFap **(F)** stained with ThT, scale bar = 10 μm. Insets: side-views of liquid cultures grown in flasks.

To confirm the differences in amyloid production, all three strains were grown on agar plates containing the amyloid-binding dye Congo Red. On such agar plates, amyloid-producing strains can be identified based on their red-brown coloration caused by the binding of Congo Red as well as their opaque or agglutinated appearance resulting from cell aggregation. Gratifyingly, only pFap showed the characteristic red-brown color indicative of amyloid formation, in contrast to WT and Δ*fap*, which showed the green color characteristic of amyloid-free *Pseudomonas fluorescens* (Figures 1A–C). In addition, pFap yielded smaller colonies as a result of reduced mobility caused by the production of the aggregative amyloid and to a minor extent the increased growth cost due to the overexpression of the amyloids. The phenotype difference was further confirmed by growth in liquid culture, where WT and Δ*fap* led to a homogeneous cloudy suspension of bacteria while pFap flocculated and appeared as a clear solution with biofilm accumulating on the sides of the growth vessel (Figures 1D–F).

### Fap amyloid confers resistance against desiccation according to AFM

**Figure 2:**
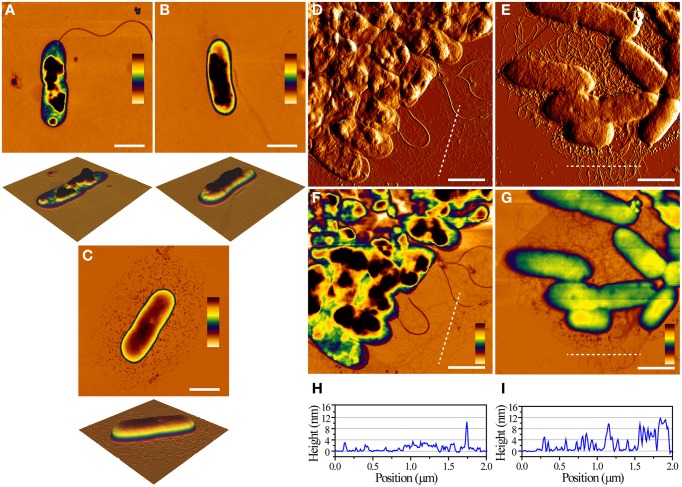
**AFM images of single cells and cell aggregates**. Height images of WT (**A**, height scale = 104 nm), Δ*fap* (**B**, height scale = 122 nm), and pFap (**C**, height scale = 213 nm) cells, scale bar = 1 μm. Error images of WT **(D)** and pFap **(E)** aggregates. Corresponding height images of WT **(F)** and pFap **(G)** aggregates, scale bar = 1 μm, and line profiles of WT **(H)** and pFap **(I)** height images marked by dashed lines.

We next imaged the individual cells using Atomic Force Microscopy. It was very straightforward to obtain and visualize individual WT and Δ*fap* cells due to their low degree of intercellular contacts, while pFap cells had to be sonicated briefly to dissociate the cellular clumps. We consistently observed that both the WT and Δ*fap* cells collapsed after brief drying in air, while pFap cells were able to maintain the rod-like shapes seen for bacteria in solution (Figures 2A–C). This difference in cell integrity was also observed when analyzing clumps of cells of WT and pFap (Figures 2D–I). Flagella are easily visible for both strains, while extracellular fibrils are clearly much more prominent for pFap. The height profiles show that pFap forms fibrils that are much higher and wider than those of WT and tend to aggregate to form larger co-existing bundles, consistent with the cells' pronounced tendency to flocculate. Overall there was little morphological difference between WT and Δ*fap* in these studies, in good agreement with their shared inability to make detectable amounts of amyloid and flocculate. Consequently we concentrate on WT rather than Δ*fap* in the following sections. pFap's ability to resist collapse upon drying, on the other hand, indicates a high level of structural robustness that is presumably conferred by the amyloid.

### AFM force spectroscopy on single cells reveals that fap amyloid induces different cellular adhesion patterns

**Figure 3:**
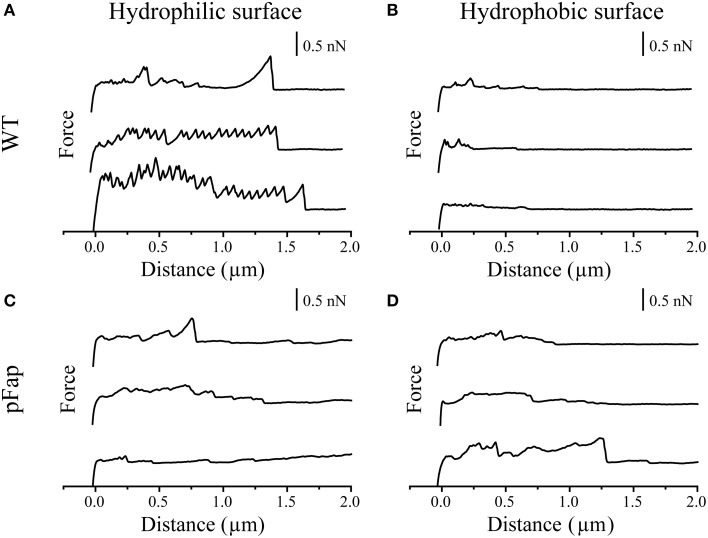
**AFM force curves of colloids with different surface properties on single cells. (A)** Hydrophilic surface on WT; **(B)** Hydrophobic surface on WT; **(C)** Hydrophilic surface on pFap; **(D)** Hydrophobic surface on pFap. Only retraction parts of the force curves are shown.

To better understand the adhesion forces conferred by amyloid on the surface of *Pseudomonas* sp. UK4, we investigated to what extent cells with and without amyloid adhered to hydrophilic and hydrophobic probes. These were constructed by gluing a 5-μm silica colloid onto the very tip of the cantilever, curing the glue with UV and functionalizing the probe surface to make it hydrophilic (UV-oxidation) or hydrophobic (silanization). Individual cells were then immobilized in an evenly distributed pattern onto a polydopamine surface and were exposed to contact with the colloid probe. The probe first approaches each cell and is then retracted while the associated cantilever deflection and movement is recorded. These data are then converted to force and probe-sample distance and plotted into force curves. The retraction force curves are shown in Figure 3 for both WT and pFap.

The sawtooth-like adhesion peaks of WT on hydrophilic surface can probably be assigned to the unfolding of the protein adhesin LapA, which is important for adhesion and surface attachment of many *Pseudomonas* species, in particular those belonging to the *P. fluorescens* and *P. putida* group (Duque et al., 2013). Accordingly, sequence analysis suggests that *Pseudomonas* sp. UK4 has a *lapA* homolog. LapA is a giant protein containing tandem repeats whose individual unfolding during retraction leads to the characteristic saw-tooth pattern, as previously reported (El-Kirat-Chatel et al., 2014). For WT, these peaks are absent on a hydrophobic surface due to different interaction modes of LapA with hydrophobic surfaces, as reported earlier (El-Kirat-Chatel et al., 2014). The absence of these saw-tooth-like peaks from force curves of pFap on hydrophilic surfaces suggests that the pFap cells are covered by other surface molecules (i.e., amyloid), thus shielding the interactions from LapA, similar to the effect of capsular polysaccharides on the short-range adhesion Ag43 in *E. coli* (Schembri et al., 2004).

The force curves directly provide the maximal adhesion force (the highest point in the retraction curve) and the final rupture length (the longest separation length at which the cell ruptures from the probe). As summarized in Table 2, the average values suggest that both the maximal adhesion force and rupture length of pFap are larger than those of WT on the hydrophobic surface. However, likely because LapA contributes to the adhesion, WT adheres more strongly than pFap to hydrophilic surfaces. Note that the errors on these measurements are considerable despite measurements on a considerable number of samples, and the differences are not statistically significant. This is remedied when looking at collections of cells in biofilm rather than the individual cells (see below).

**Table 2:** **Maximal adhesion force and final rupture length of WT and pFap cells on hydrophilic and hydrophobic surfaces^a^**.

**Surface type**	**WT**		**pFap**	
	**Max. adhesion force (nN)**	**Final rupture length (μm)**	**Max. adhesion force (nN)**	**Final rupture length (μm)**
Hydrophilic	0.61 ± 0.32	1.07 ± 0.45	0.30 ± 0.17	1.38 ± 0.76
Hydrophobic	0.32 ± 0.17	0.70 ± 0.47	0.50 ± 0.26	1.40 ± 0.82

a *Based on data shown in Figure 3*.

### Fap amyloid enhances adhesion to spherical probes, especially those with hydrophobic surfaces, and increases the contact angle of water droplets

**Figure 4:**
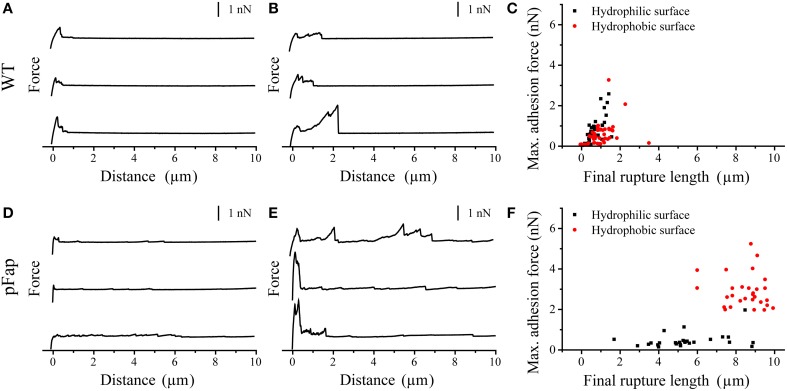
**AFM force curves of colloids with different surface properties on cell aggregates. (A)** Hydrophilic surface on WT; **(B)** Hydrophobic surface on WT; **(C)** Max. adhesion force vs. final rupture length of the force curves on WT; **(D)** Hydrophilic surface on pFap; **(E)** Hydrophobic surface on pFap; **(F)** Max. adhesion force vs. final rupture length of the force curves on pFap. Only retraction parts of the force curves are shown.

The situation became considerably clearer when we instead turned to force spectroscopy measurements on cell aggregates (Figure 4). We did not observe any saw-tooth patterns in the force curves on biofilm, indicating that all cells in biofilm are completely covered by EPS. EPS are therefore the major components which contribute to the adhesive properties of biofilm. For WT, rupture lengths and maximal adhesion forces were uniformly small and not statistically different for the two types of surfaces (Table 3). In contrast, pFap shows much longer rupture lengths on both surfaces than WT does, and the maximal adhesion force is 5-fold increased compared to WT on hydrophobic surfaces (but not on hydrophilic surfaces). We see a large variation in the final rupture length particularly for pFap on hydrophilic surfaces. We attribute this to the fact that EPS in biofilm are composed of polymers of various lengths. At each measurement random molecules or molecular assemblies are picked up by the probe at random positions along the molecules, leading to varying final rupture length. While EPS is present in biofilm for both WT and pFap, it is possible that interactions between amyloid, other EPS components and the hydrophilic surface lead to a particularly large variation.

**Table 3:** **Maximal adhesion force and final rupture length of WT and pFap cell aggregates on hydrophilic and hydrophobic surfaces^a^**.

**Surface type**	**WT**		**pFap**	
	**Max. adhesion force (nN)**	**Final rupture length (μm)**	**Max. adhesion force (nN)**	**Final rupture length (μm)**
Hydrophilic	0.707 ± 0.616	0.653 ± 0.368	0.473 ± 0.365	5.430 ± 1.762
Hydrophobic	0.524 ± 0.566	0.822 ± 0.863	2.891 ± 0.819	8.486 ± 1.007

a *Based on data shown in Figure 4*.

**Figure 5:**
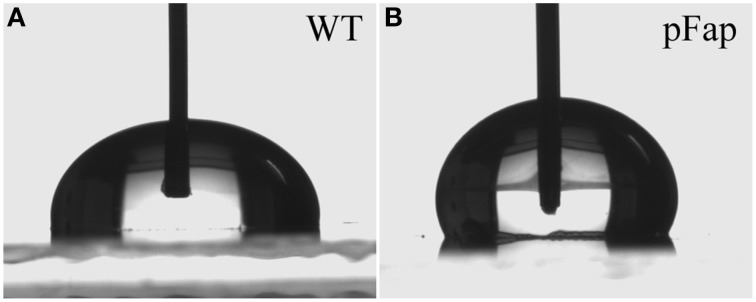
**Video snapshots from contact angle measurements of (A) WT and (B) pFap**.

The high adhesion forces and increased rupture lengths for pFap on hydrophobic surfaces suggested that the biofilm might be more hydrophobic itself. We confirmed this by measuring the contact angle formed when water drops are deposited on cell lawns (Figure 5). Water will try to minimize contact with a hydrophobic surface, so the larger the contact angle, the more hydrophobic the surface. Due to the instability of static water drops on porous and absorptive cell lawns, we had to carry out dynamic measurements where contact angles were recorded over time. Contact angles at the critical points where water drops spreads to make contact with new surfaces were defined as advancing contact angles. The advancing contact angles of WT and pFap were 95.2 ± 1.1 and 128.1 ± 0.6°, respectively. The measured contact angle may overestimate the actual contact angle of the bacterial surface, because the micro scale porosity of the bacterial lawn could lead to an increase in the contact angle. However, the data clearly suggest that pFap is more hydrophobic, in good agreement with our AFM measurements.

### AFM nanoindentation measurements reveal that fap amyloid increases biofilm stiffness 20-fold

**Figure 6:**
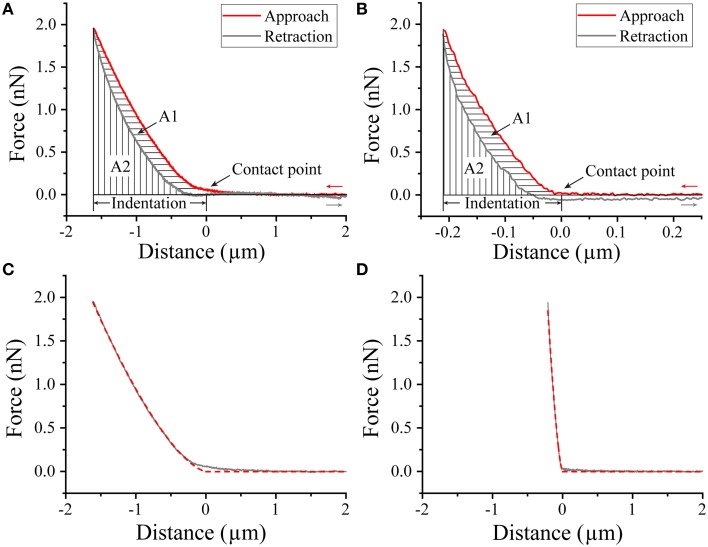
**AFM Nanoindentation of biofilm**. Approach-retraction force curves on WT **(A)** and pFap **(B)** biofilm illustrating contact point, indentation depth, and plastic deformation (A1) and elastic recovery (A2). Indentation curves (approach part of the force curves, solid lines) and fit to Hertz model (dashed lines) for WT **(C)** and pFap **(D)**.

To gain insight into the mechanical properties of the biofilm, we carried out nanoindentation experiments, in which a large microbead was pressed into the bacterial biofilm using the AFM cantilever (Figure 6). During the approach of the probe, a repulsive force is generated immediately after the bead establishes contact with the biofilm, and the approach continues until a repulsive force of ~2 nN is reached, after which the the probe is retracted. The approach and rectraction curves do not overlap completely, because deformation of biofilm is not completely elastic, i.e., there is a certain degree of plastic deformation that occurs. This can be quantified by the plasticity index ψ_P_ (Equation 2). Plasticity indices ψ_P_ of WT and pFap were 0.31 ± 0.03 and 0.33 ± 0.02, respectively (*P*>0.05, *df* = 6), indicating that they have similar plastic deformation. However, the indentation depth under the same load (2 nN) was much larger for WT (1.540 ± 0.047 μm) than for pFap (0.238 ± 0.025 μm), indicating that the pFap film is much stiffer. The indentation curves can be further fitted to the Hertz model of contact mechanics to provide Young's modulus, which is a measure of biofilm stiffness. Indeed, the fitted Young's modulus of pFap (2.01 ± 0.08 kPa) is 20 times as high as for WT (0.10 ± 0.01 kPa).

### All fap genes except FapA contribute to biofilm stiffness

**Figure 7:**
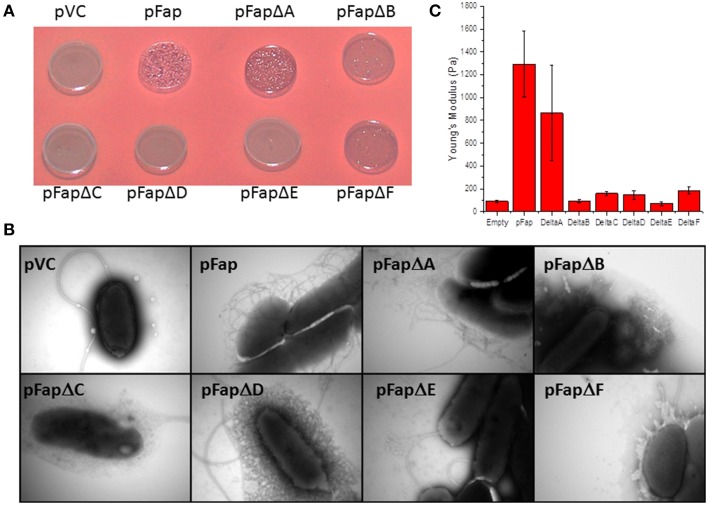
**Analysis of the effect of removing individual genes from the ***fap*** operon**. Δ*fap* was transformed with the empty plasmid (pVC), a plasmid containing the complete *fap* operon (pFap) or plasmids containing the *fap* operon with the individual *fap* genes deleted. **(A)** Congo Red binding. Cells were grown on CFA agar plates containing Congo Red. A dark red color indicates amyloid formation. **(B)** Transmission electron microscopy images of cells taken from cell cultures at stationary phase. **(C)** Indentation experiments showing the value of Young's modulus for biofilms grown with the different mutants.

To evaluate the contribution of individual protein components to the biofilm, we constructed plasmids containing *fap* operons missing each one of the six genes in turn. We then evaluated how these individual deletions affected Congo Red binding, fibril formation and biofilm stiffness. The only deletion mutant which produced the classical amyloid positive colony morphology on the Congo Red agar plates was pFapΔA (Figure 7A). Since Congo Red is not able to penetrate the intact bacterial outer membrane (Sleytr et al., 1988), binding implies successful export of amyloid for pFapΔA. pFapΔB and pFapΔF produced transparent colonies that bound Congo Red weakly. The weak binding of Congo Red by pFapΔB and pFapΔF are likely caused by non-amyloid components, probably polysaccharides. It has previously been shown that overexpression of the *fap* operon in *P. aeruginosa* can induce alginate production (Herbst et al., 2015) and this effect may be independent of FapB and FapF. The remaining deletion strains displayed morphologies similar to Δ*fap*.

The consequences of the deletion of these genes become clearer when individual cells are visualized by TEM. Deletion of all individual genes except *fapA* significantly reduces the amount of *bona fide* curly fibrillary outgrowths from *Pseudomonas* (Figure 7B). Only pFapΔA shows curly structures comparable to (though less dense than) those of pFap. pFapΔC has a halo of fuzzy structures which cannot be amyloid (cfr. Figure 7A), and pFapΔD has a mesh-like cover that likely represents excreted vesicles. These differences are strikingly confirmed by biofilm stiffness measurements (Figure 7C), which reveal that only the pFapΔA mutant leads to biofilm with a stiffness comparable to that of the intact *fap* operon; all other constructs show stiffness levels comparable to that of the empty vector. Note also that the pFapΔA mutant leads to overproduction of FapB at the expense of FapC (Dueholm et al., 2013a), explaining the change in appearance of the cells in Figure 7B.

## Discussion

### Role of amyloid in the mechanical robustness of biofilms

We have established that functional amyloid expression in *Pseudomonas* significantly affects cell properties, such as ability to withstand drying, hydrophobicity and biofilm rigidity. It cannot be ruled out that these effects are linked to secondary changes induced by overexpression of amyloid, such as altered expression patterns of other components in the EPS. A recent proteomic study (Herbst et al., 2015) showed that *fap* expression led to upregulation of alginate biosynthesis, turning the *P. aeruginosa* PAO1 into a mucoid phenotype. While mucoid biofilms are thicker, rougher and more antibiotic-resistant than the non-mucoid versions which are flat and dense (Hentzer et al., 2001), their impact on biofilm stiffness is unclear and might intuitively be expected to lead to a more expanded structures. Our data make it clear that amyloid expression and formation on the surface is linked to major changes in cell robustness. Furthermore, deletion of individual members of the *fap* operon (which is not expected to alter the cellular response to *fap* expression significantly) completely removes this increased biofilm stiffness. We have previously reported that *Pseudomonas* amyloid is able to bind small metabolites (Seviour et al., 2015), and this binding ability correlates with metabolite hydrophobicity. This strongly suggests that amyloid in itself is hydrophobic and provides a straightforward driving force for intercellular contacts. It also highlights the role of amyloid in providing high elastic strength and thus mechanical robustness to the biofilm. We speculate that this property is conferred because the relatively stiff amyloid fibrils are embedded within a more flexible matrix provided by eDNA and polysaccharides, analogous to the amyloid-amorphous phase combination seen in spider dragline silk (Hagn, 2012) or even reinforced concrete (steel wires within the concrete matrix). Increased protein content in the EPS has also been associated with increased biofilm strength (Pellicer-Nácher and Smets, 2014) in a study of nitrifying membrane-aerated biofilms, though in this case the proteins rendered the biofilm more hydrophilic.

It is clear that increased biofilm strength requires production of a substantial amount of amyloid. Although the mutants lacking FapB and FapF showed some level of Congo Red binding, the overall production of extracellular amyloid had clearly been reduced, and correspondingly the biofilm stiffness was just as low as that of the Δ*fap* mutant. Based on its sequence homology to FapC, we have proposed that FapB is a nucleator for amyloid formation on the outer membrane surface (Dueholm et al., 2010), analogous to CsgB (Hammer et al., 2007). Sequence comparisons also suggest that FapF provides a conduit for transport of FapC (and FapB) through the outer membrane. Thus, removal of these two components should be definitely reduce FapC export and formation of the amyloid state on the bacterial cell surface. It remains more mysterious why deletion of FapE (a minor component of amyloid) and FapD (a putative peptidase) should completely abolish amyloid production, and further studies are clearly needed to delineate their specific contributions to the production of amyloid. In contrast, the removal of FapA does not compromise the overall function of the *fap* operon, but largely seems to affect the balance between FapC and the other amyloid component FapB which is predicted to be a fibril nucleator (Dueholm et al., 2013a) but can also fibrillate well on its own *in vitro* (B.S.V. and D.E.O., unpublished).

We note that the major component in *Pseudomonas* amyloid, the protein FapC, consists of 3 imperfect repeats connected by linkers of variable length (Dueholm et al., 2010). The repeats likely constitute the amyloid core since their removal completely abolishes FapC's ability to fibrillate (B.S.V. and D.E.O., unpublished), while the linkers show variable length but largely contain hydrophilic residues. It is possible that the hydrophobicity of the biofilm, as well as the interactions with other EPS components, may be modulated by the composition and length of the linkers. This in turn may also affect the mechanical properties of the ensuing biofilm. Our nanoindentation assay provides a very convenient tool to assess this. Given the important role of mechanical forces between bacterial cells in the early build-up of biofilm (Grant et al., 2014), amyloid can also play a role in promoting rapid establishment of biofilm. We have already observed this in a number of *Pseudomonas* strains overexpressing different variants of the *fap* operon, which led to more rapid surface colonization and alterations in biofilm morphology (Dueholm et al., 2013a).

### Measurement of hydrophobicity by contact angle

Static contact angle measurement has been used by researchers to characterize the hydrophobicity of bacterial cells (Busscher et al., 1984; Fernández et al., 2007; Seale et al., 2008; Gallardo-Moreno et al., 2011). We found it difficult to obtain consistent results across different replicates using this measurement, probably due to the fast penetration of water droplets into the bacteria lawns. We instead used advancing contact angle measurement for determining hydrophobicity (Liu et al., 2008), which yielded more reproducible contact angles. However, caution must be taken to use contact angles on bacterial lawns as a direct measurement of hydrophobicity. As pointed out by a recent report investigating this complex topic (Gallardo-Moreno et al., 2011), contact angles on dried bacterial lawns are so strongly dependent on measuring time and environmental conditions that an absolute measurement of hydrophobicity and Gibbs energy of bacterial cell surfaces is essentially impossible. Nevertheless, the method is still useful as an indication of relative hydrophobicity, especially when all measurements are done using the same procedure.

### Colloidal probes as a tool to determine biofilm mechanical properties

Since its invention in the early 1990s (Butt, 1991; Ducker et al., 1991), AFM force spectroscopy with colloidal probes has been widely used to measure the mechanical properties of soft biological materials (Rosenbluth et al., 2006; Sokolov et al., 2007; Plodinec et al., 2012). Compared to the conventional sharp AFM tips, colloidal probes have more well-defined contact areas, and they are therefore recommended for measurement when spatial resolution is not of concern (Dimitriadis et al., 2002; Loparic et al., 2010). Penetration of soft materials can also be avoided by colloidal probes. In the current study, relatively large microbeads were selected for nanoindenation measurements primarily because large contact area provides averaging of the mechanical properties, that is, the global instead of local mechanical properties are measured (Loparic et al., 2010). This eliminates large variation of the results as the biofilm is usually highly heterogeneous. This is confirmed by large data variation from indentation with 5 μm colloid probe (data not shown). Besides, the Hertz model for spherical indenter used to calculate the mechanical properties of the sample, assumes that the contact radius is less than 10% of the indenter radius. This assumption cannot be met when using a small sphere on a soft sample which usually results in a large indentation depth for biofilm samples, even with small indentation forces.

Biofilm elasticity measured by colloidal probe indentation is in the current study within the range of 0.017–170 kPa reported previously using different techniques (Stoodley et al., 1999, 2001; Körstgens et al., 2001; Lau et al., 2009). The wide range of biofilm elasticities is due to the inherent differences among biofilm matrix composition in various bacterial strains as well as the growth conditions, sample preparation, and different methodologies used. One needs to be careful in comparing results using different techniques. A most relevant case to our study is that Casey and co-workers recently used AFM colloidal probe to measure the elastic modulus of *P. fluorescens* PCL1701 biofilm, and reported mean elastic moduli of 33 ± 22 kPa and 2.7 ± 1.3 kPa for calcium-free and calcium-supplemented biofilm, respectively (Safari et al., 2014). Moreover, they also detected a softer surface layer of the calcium-supplemented biofilm with an elastic modulus of 0.39 ± 0.24 kPa up to an indentation depth of 1.27 ± 0.33 μm. This data is comparable to our measurement on WT, which should not be surprising because our CFA growth medium contains divalent ions of magnesium and manganese.

The roles of amyloids as biofilm structural components have been demonstrated in several species. Curli amyloids are essential for *Escherichia coli* (Vidal et al., 1998) and *Salmonella enteritidis* (Austin et al., 1998) to attach to the surface and form thick biofilm. Amyloid fibers formed from TasA protein in *Bacillus subtilis* have also been shown to be required for the formation of robust pellicle biofilms, while aggregation of phenol-soluble modulins protect *S. aureus* biofilm against mechanical and enzymatic attack (Schwartz et al., 2012). We have previously shown that Fap amyloids facilitated *Pseudomonas* biofilm formation (Dueholm et al., 2013a). All these studies, however, are limited to morphological investigation. The study we present here is, as far as we know, the first to investigate the effect of amyloids on biofilm mechanical properties in a quantitative way.

### Robust biofilm as a key to successful colonization?

The extraordinary mechanical robustness that functional amyloids provide to bacterial biofilms raises the question of their role in biofilm survival in different contexts. *Pseudomonas fluorescens* is non-pathogenic and ubiquitous in the environment where it successfully colonizes inorganic or biological (e.g., plants) surfaces in, e.g., soil and aquatic environments. Other *Pseudomonas* species are also wide spread. One might speculate that the functional amyloid of *P. fluorescens* and other *Pseudomonas* species is one of the keys to the robustness required to successfully colonize such diverse habitats. Future studies will no doubt reveal new insights into the role of functional amyloids in the ecology of bacteria, and how amyloid production in biofilm affect the biofilm's resistance to environmental stressors, grazing predators, or production of antimicrobial compounds from competing microorganisms.

### Conflict of interest statement

The authors declare that the research was conducted in the absence of any commercial or financial relationships that could be construed as a potential conflict of interest.

## Abbreviations

AFM: Atomic Force Microscopy
EPS: extracellular polymeric substances
Fap: Functional amyloid in *Pseudomonas*
D*fap*: *Pseudomonas* strain lacking the *fap* operon
pFap: *Pseudomonas* strain transformed with a plasmid overexpressing *fap*
WT: wildtype.
